# Cases from Practice

**Published:** 1893-10

**Authors:** 

**Affiliations:** Hamburg, Germany


### CASES FROM PRACTICE.

### Dr. Hesse, Hamburg, Germany.

[From the *Archiv für Homœopathie.* Translated by A. McNeil, San Francisco.]

A female teacher, who was treated successfully in April, 1890, with Causticum for a rheumatic affection of the left arm and right leg, came to me on the 20th of August, 1891, with psoriasis on both hands, which had existed three months. She also had hot hands and flying heat in the face. Sepia$^{30}$, a powder every week.

December 9th, 1891, she reported that she only took one-half of the six powders I gave her. She is now complaining of a gastric disorder, worse when sitting bent over and in the morning—weakness. Relying on the previous good effect of Sepia, and as there was nothing in the present symptoms to the contrary, I gave it again.

December 13th she reported considerable improvement.

A tax official, thirty years old, had long suffered from a dry itching eruption on the back of his hands. Six years ago had an itch-like eruption on the body, which was treated homœopathically. April 7th, 1891, Sepia$^{30}$, a powder every week.

He returned November 18th, 1891, on account of an acute trouble, which required Pulsatilla. His eruption had disappeared.

April 11th, 1892, he came again on account of a light attack of gonorrhœa, which had existed eight days, no other symptoms. As I considered that Sepia was his constitutional remedy, I gave it to him once more in the 30th, five powders, one each evening.

May 7th the discharge decreased, the old eruption reappeared on the back of his hands after taking the powders. But it has now disappeared, showing how suitable Sepia was for his constitution and how safely that remedy acts. I gave him Placebos and he did not again return.

Sepia is in general one of the most important remedies in eruptions and tetter. It stands in close relation to the skin of the hands, and more particularly to their dorsal surfaces. Von Bœnninghausen gives three remedies the highest rank to the back of the hands—viz.: Natrum-carb., Rhus, and Sepia in the second are Calc-carb., Kreos., Sambucus, and Sulphur.

Farrington says Sepia is the most important remedy for small ulcers, particularly those on the wrists. In dry tetter of the backs of the hands this remedy has proved so useful that I always give it unless the symptoms indicate another remedy. For the face of the hands Sepia only takes the third rank. In that peeling off of the palms, which I have often seen as the result of lues, Sepia has been useful. Jahr gives as one of its symptoms, "Peeling off of the skin of the palms of the hands, leaving round bright red spots on the ball of the right thumb, with violent itching, which is not better by scratching."

Von Bœnninghausen recommends for white crusted tetter on the hands, Anacard., Sepia, and Thuja.

In chapped hands, where there are deep, bleeding cracks in the skin, an affection often worse in winter, and not accompanied by any other symptoms, Petrol. has been for me the most useful remedy. I give it almost exclusively in the 30th potency, one dose a week. However, I recall one case in which I gave it successfully several years in the 3d. [Wouldn't the 30th and higher have cured it in much less time?—McN.]

### Cases Cured by Dr. Wassely Keil, Germany.

In the evening of June 27th, 1892, I was called in haste to see a woman. She was forty-four years old, and her periods had continued fourteen days. Her menses had been absent eight weeks. To-day she had felt particularly well, and called on a friend, in whose garden she was attacked by a violent uterine hemorrhage. She was hurried to her home in a carriage. It was thought to be a threatened abortion. I found her in bed *by an open window*, warmly covered, in *great anguish.* She was constantly passing *dark blood in large clots,* worse by every movement. She was not in pain. While her habit was to lie with her head low, *she now wanted to lie with her head high,* and preferred the dorsal position. On preceding days *she had much flatulence.*

I gave her Lycop.[30], two globules, dry, on her tongue.

In two hours her husband reported that immediately after taking the medicine a large clot passed and then ceased. She continued to improve. No more medicine.

